# Investigation of Effects of Cushioning Packaging on the Physiological and Quality Changes in Chinese Olive Fruits During Cold Chain Transportation

**DOI:** 10.3390/foods13244133

**Published:** 2024-12-20

**Authors:** Han Lin, Fanghao Fu, Jinghai Li, Jiahui Liu, Kaiyang Du, Bingxia Zhu, Zhixiong Guo, Tengfei Pan, Wenqin She

**Affiliations:** College of Horticulture/Institute of Storage, Transportation and Preservation of Horticultural Products, Fujian Agriculture and Forestry University, Fuzhou 350002, China; linhan_haha@163.com (H.L.);

**Keywords:** Chinese olive fruits, cushioning packaging, antioxidant enzyme activities, metabolome analysis, shelf quality

## Abstract

To gain a deeper understanding of the mechanisms by which cushioning packaging preserves the quality of Chinese olive fruits during cold chain transportation and extends their shelf life, this study simulated cold chain conditions and investigated the effects of cushioning packaging on the physiology, antioxidant capacity, and secondary metabolites of fruits during a 20-day shelf life. The results indicated that the decay rate in cushioning-packaging-treated fruit was 75% lower than that in the unbuffered packaging fruit at day 20 of shelf life. Simultaneously, cushioning packaging treatment mitigated the damage severity of the cell membrane structure and kept the cell membrane permeability at a low level, which was 15.34% lower than that in the unbuffered packaging fruit at day 20 of shelf life. Additionally, cushioning packaging effectively restrained the increases in malondialdehyde (MDA) content and alleviated the decline in chlorophyll and total flavonoid contents. It kept a balance among reactive oxygen species (ROS), antioxidant levels, and antioxidant enzyme activities, thereby reducing mechanical-damage-induced decay rates in Chinese olive fruits during the shelf life. Furthermore, metabolome analysis of Chinese olives during the shelf life was performed comparing those without buffered packaging to those with buffered packaging. The metabolome analysis found that the flavonoid biosynthetic pathway exhibited a higher accumulation of chrysin, neohesperidin, naringenin chalcone, sakuranetin, quercetin, catechin, and naringenin metabolites in cushion-packaging treatment compared to those without cushioning treatment. Furthermore, within the phenylalanine metabolic pathway, the accumulation of phenylalanine, *p*-coumaraldehyde, *p*-coumaric acid, coniferin and caffeoyl quinic acid metabolites was significantly higher in buffered-packaging groups compared to those without buffering. Together, these findings suggest that cushioning packaging can effectively sustain the integrity of cell membranes and enhance the shelf-life quality of Chinese olive fruits by regulating the balance of ROS and mitigating oxidative stress during cold chain transportation.

## 1. Introduction

Chinese olive (*Canarium album* Lour.), affectionately known as ‘Bailan’ or ‘Qingguo’, is a distinctive fruit with dual uses as medicine and food in southern subtropical China [1]. The fresh Chinese olives, with their benefits of quenching thirst, stimulating appetite, strengthening the spleen, facilitating digestion, and eliminating bad breath, are highly esteemed by consumers. However, fresh Chinese olives are highly susceptible to mechanical damage during storage and transportation, which further leads to the degradation of storage quality [2]. Hence, it is extremely crucial to employ suitable packaging methods to reduce damage in Chinese olives during transportation to extend their shelf life.

Air column bag cushioning packaging material, which is known for its outstanding air tightness and moisture-proof performance, can offer professional packaging cushion protection for fruits and vegetables, effectively preventing bumps and compressions during transportation, thereby reducing damage [3]. Some studies have shown that packaging plays a crucial role in preventing fruits from being damaged by vibration during the cold chain logistics process. Chaiwong et al. [4] evaluated the vibration damage on guava peels through digital image analysis and found that a novel eco-friendly cushioning material, NRL-FN, provides better buffering performance against vibration bruising. Foam-net packages provided essential cushioning protection in the transportation of harvested Huanghua pears, thereby delaying fruit softening and preserving superior quality [5].

Fruit appearance and fruit weight were the most important quality parameters, determining fruit acceptability by consumers [6]. Mechanical damage can result in physical and physiological changes in fruit tissues, leading to an elevated decay rate and water loss rate, along with the accumulation of MDA [7,8]. Meanwhile, it can cause defects in the fruit appearance. Among them, appearance defects are closely related to pigments, while the discoloration of postharvest fresh fruit is related to the reduction of pigments (containing chlorophyll and the total flavonoid content), which dramatically influence their storage and market acceptance after the harvest [9]. Furthermore, the postharvest quality of fruits and vegetables is closely related to the level of ROS [10]. Antioxidant enzymes, such as catalase (CAT) and ascorbate peroxidase (APX), can protect the cellular membrane integrity from oxidative damage caused by high ROS generation to fruit cell macromolecules [11]. The research demonstrated that figs treated with 1-MCP in conjunction with modified atmosphere packaging (MAP) retained higher ROS-scavenging enzyme activities, significantly inhibiting the decay of figs and maintaining higher quality during cold storage as compared to figs without MAP [12,13]. Litchi fruits in MAP bags can sustain higher antioxidant enzyme activity during cold storage, thereby delaying postharvest peel browning and preserving their quality [14]. In general, appropriate packaging has important roles in maintaining the activity of antioxidant enzymes of fresh produce [15].

The decay of Chinese olive fruits during shelf life has been attributed to cold injury [16], pathogen infection [17], and other factors. All of these elements disrupt cellular compartmentalization, enabling polyphenol oxidase (PPO) in chloroplasts and other plastids to interact with phenolic substrates in the vacuole, ultimately resulting in a decline in fruit quality [18]. Peroxidase (POD) may also play a role in this process [19]. In addition, there is a close relationship between fruit deterioration and metabolism disorders of membrane lipids. Lipoxygenase (LOX), as a vital lipid enzyme, is involved in membrane damage leading to the dysregulation of membrane lipid metabolism, thereby reducing postharvest quality [20].

Recent metabolomic technology has provided comprehensive information regarding the similarities and differences in metabolite compositions between samples, allowing us to analyze the intricate regulatory process of postharvest fruit physiology from the perspective of metabolic pathways or regulatory networks. For instance, systematic research on the metabolic components of different tissues in blackberry was investigated through nontargeted LC–MS metabolomics, thereby analyzing the differences in the accumulation of flavonoids in various blackberry tissues [21]. Metabolomic studies were conducted on postharvest citrus to investigate the pathogenesis of green mold disease [22]. Therefore, understanding the impact of cushion packaging on the metabolic pathways of Chinese olives during cold chain transportation can provide deeper insights into optimizing their cold chain transportation packaging solutions.

At present, post-harvest commercial treatment and storage are in a relatively weak position within the whole industrial chain. In addition, the focus of research hotspots in the postharvest preservation technology of Chinese olives is cold storage [23] and fresh-keeping agents [24]. There are few studies on the buffer packaging of Chinese olive fruit during cold chain transportation. In this study, we investigate the impacts of cushion packaging on postharvest quality, the content of antioxidant substances, and antioxidant enzymes during cold chain transportation during the shelf life of Chinese olive fruits. In addition, metabolomic analysis was conducted on the metabolome to investigate the impact of packaging under cold chain transportation on the metabolic pathways of Chinese olives during the shelf life. The findings of this work will offer new knowledge for the effects of packaging on the quality of Chinese olive fruits during cold chain transportation and provide theoretical references for their transportation and shelf life.

## 2. Materials and Methods

### 2.1. Chinese Olive Fruits and Treatment

The ‘Meipu 2’ Chinese olives at a commercial mature stage, with an L* value of 45–55 and a TSS content of 8–9%, were harvested from the White Stone Pit Jiuyuan Chinese olive Professional Cooperative situated in Minqing County, Fuzhou, China. Medium-sized, pest-free, and undamaged Chinese olives were harvested from six trees exhibiting uniform growth. Approximately 80 kg of fruits was utilized for the experiment. Following harvest, the fruits underwent a thorough wash with clean water and then were allowed to air-dry naturally. Subsequently, the selected fruits were categorized into three groups; each group comprised three boxes containing six bags per box, with each bag weighing 500 g. The outer packaging was standardized as aluminum film cold chain carton boxes and the inner packaging consisted of polyethylene film bags measuring 0.08 mm in thickness. One group received cushioning treatment using air column bags (CctQ), and another group lacked any cushioning (Cct) (Figure S1). Both samples were transported to the laboratory via a cold chain truck. Additionally, there was a control group consisting of Chinese olives that had not been transported. Then, a simulated shelf-life study lasting for 20 days at 90% RH and 9 ± 1 °C was conducted on these three fruit groups. Samples were taken with a sterile paring knife and then frozen in liquid nitrogen and subsequently stored at –80 °C for further analysis.

### 2.2. Evaluation of the Decay Rate and Weight Loss

The decay rate was defined following the approach of Wu et al. [25] with some modifications. For that, 100 Chinese olives were observed, and the decay rate was calculated as the ratio of decayed fruit to the total number of observed fruits. Results were expressed in percentages.

To evaluate the weight loss of olives during shelf life according to the method of Liang et al. [26], the percent of weight loss was computed by comparing the weight of 30 Chinese olives on storage day 0 (harvest day) with the weight of 30 Chinese olives on the different days of storage.

### 2.3. Assessment of Chlorophyll and Total Flavonoid Content

The approach of Zhang et al. [27] with minor revisions was utilized to conduct the chlorophyll content determination. Chinese olive pulps (1.0 g) from ten fruits were ground with extraction medium (containing 90% ethanol and 80% acetone) and then left to stand for 7 h in a dark environment. The above reaction mixture was centrifuged to measure the absorbance at 663 nm, 646 nm, and 470 nm. The results were expressed as mg g^−1^.

The total flavonoid content was determined following the protocol of Zhang et al. [28] with slight modifications, adding 70% ethanol solution to 1 g of frozen Chinese olive pulp taken from ten fruits as the extract solution for the follow-up assay of the total flavonoid content. These solutions were treated by an incubation at 50 °C for 1.5 h and a centrifugation (8000 rpm) for 10 min. After that, the solution was diluted with ethanol to a final volume of 25 mL. The reaction system to assay the total flavonoid content consisted of 1 mL of supernatant and 1 mL of ethanol. Subsequently, sodium nitrite solution, aluminum nitrate solution, and sodium hydroxide solution were added to the mixture liquids every 6 min in sequence. After rapid mixing, the reaction system was incubated for 10 min, and the absorbance of this solution was measured at 514 nm. The unit was denoted as % based on the catechin equivalents.

### 2.4. Measurement of MDA Content and Cell Membrane Permeability

One gram of the flesh tissue of ten fruits was randomly selected to determine the MDA content according to the protocol of Si et al. [29] with certain modifications. The sample was homogenized in 10% TCA and a small amount of quartz sand. Subsequently, the homogenate was centrifuged at 4000 rpm for 10 min. The centrifuged supernatant was mixed with 0.6% TBA solution and then subjected to a reaction in a boiling water bath for 15 min. Following rapid cooling, the reaction system was subjected to a further centrifugation. Thereafter, the absorbance of the supernatant was measured at 532, 600, and 450 nm. The results of MDA contents were expressed as μmol·g^−1^.

One gram of flesh was collected from ten fruits for the determination of cell membrane permeability using the method of Song et al. [30]. The sample was put into a beaker filled with 25 mL of deionized water, and then, it was sealed with plastic wrap. Following a 12 h settling time, the measured value of the conductivity meter is C1, and after boiling, the measured value is C2. The cell membrane permeability was represented as C1/C2 × 100%, with its unit denoted as %.

### 2.5. Measurement of AsA and GSH Contents

The contents of AsA and GSH were assayed using the methodologies of Lin et al. [17], adding 5% TCA solution to 0.5 g of frozen Chinese olive pulp as the extract solution for the follow-up assay of AsA and 10% TCA solution as the extract solution for the follow-up of GSH. These solutions were treated via vortexing and centrifugation (4000 r min^−1^) at a low temperature for 10 min.

The reaction system for assay AsA consisted of 1 mL of extract solution, 0.5 mL of dithiothreitol, 0.5 mL of PBS (pH 7.8), 0.5 mL of 20% TCA, 0.5 mL of phosphoric acid, 1 mL of 4,7-diphenyl-1,10-phenanthroline, and 0.5 mL of ferric chloride. After rapid mixing, the reaction system was incubated at 30 °C for 90 min, and the absorbance of this solution was measured at 534 nm. The result was expressed as mg·g^−1^.

The reaction system to assay GSH consisted of 1 mL of extract solution, 0.1 mL of PBS (pH 7.8), and 0.2 mL of 5, 5-dithiobis (2-nitrobenzoic acid). After rapid mixing, the reaction system was incubated at 30 °C for 5 min, and the absorbance of this solution was measured at 412 nm. The result was expressed as μg·g^−1^.

### 2.6. Determination of CAT, APX, PPO, LOX, and POD Activities

The activities of CAT, APX, PPO, LOX, and POD were determined based on the methodology described by Hou et al. [31] and Hasan et al. [32], with slight adjustments.

Here, a 0.05 mol L^−1^ PBS solution in 0.5 g of frozen Chinese olive pulp as the extract solution was added for the follow-up assay of CAT, APX, LOX, and POD. These solutions were treated via vortexing and centrifugation (8000 r/min) at a low temperature for 20 min.

The reaction system to assay CAT consisted of 0.1 mL of extract solution, 1 mL of H_2_O_2_, and 1.9 mL of PBS (pH7.8). After rapid mixing, the absorbance of this solution at 0 min and 2 min was measured at 240 nm.

The reaction system to assay APX consisted of 0.2 mL of extract solution, 2.7 mL of acetate buffer (pH 5.6), 0.1 mL of H_2_O_2_, 0.1 mL of AsA, and 0.1 mL of ethylenediaminetetraacetic acid disodium salt. After rapid mixing, the absorbance of this solution at 0 min and 2 min was measured at 290 nm.

The reaction system to assay PPO consisted of 0.1 mL of extract solution, 3.9 mL of PBS (pH 5.6), and 1 mL of pyrocatechol solution. After incubating at 37 °C for 15 min, the reaction solution was centrifugated to measure the absorbance of this supernatant at 420 nm.

Additionally, LOX activity was assayed following the instructions of the plant alternative oxidase ELISA kit (Shanghai Enzyme-linked Biotechnology Co., Ltd., Shanghai, China). The absorbance of this solution was measured at 450 nm.

The reaction system to assay POD consisted of 0.1 mL of extract solution, 2 mL of acetate buffer (pH 5.6), 0.1 mL of H_2_O_2_, and 0.8 mL of guaiacol. After rapid mixing, the absorbance of this solution at 0 min and 2 min was measured at 470 nm.

The protein amounts of CAT, APX, PPO, and POD solutions were assayed using the procedure of Bradford [33]. These enzymes were presented as U·g^−1^·min^−1^ based on the protein mass. In addition, LOX was presented as U·g^−1^ based on the protein mass.

### 2.7. Analysis of Metabolites

Chinese olive samples (50 mg) were processed using vacuum freeze-drying technology in a lyophilizer (Scientz-100F). The analysis was performed using an ultra-high-performance liquid chromatography equipped with a chromatography column using a T3 bonding technique and coupled to an electrospray-ionization quadrupole time-of-flight mass spectrometer (Waters, Milford MA, USA) [34]. PCA and orthogonal partial least squares discriminant analysis (OPLS-DA) were applied to distinguish between different sample categories and verify the reliabilities of samples. A significance threshold of a VIP ≥ 1 and *p*-value < 0.05 was employed to identify DAMs [35].

### 2.8. Statistical Analysis

Statistical analyses of the differences were performed using SPSS 27 with one-way analysis of variance (ANOVA). All experimental data were assessed in triplicate and shown as the mean ± standard error (*n* = 3).

## 3. Results

### 3.1. Changes in Storage Quality of Fruit

**Figure 1:**
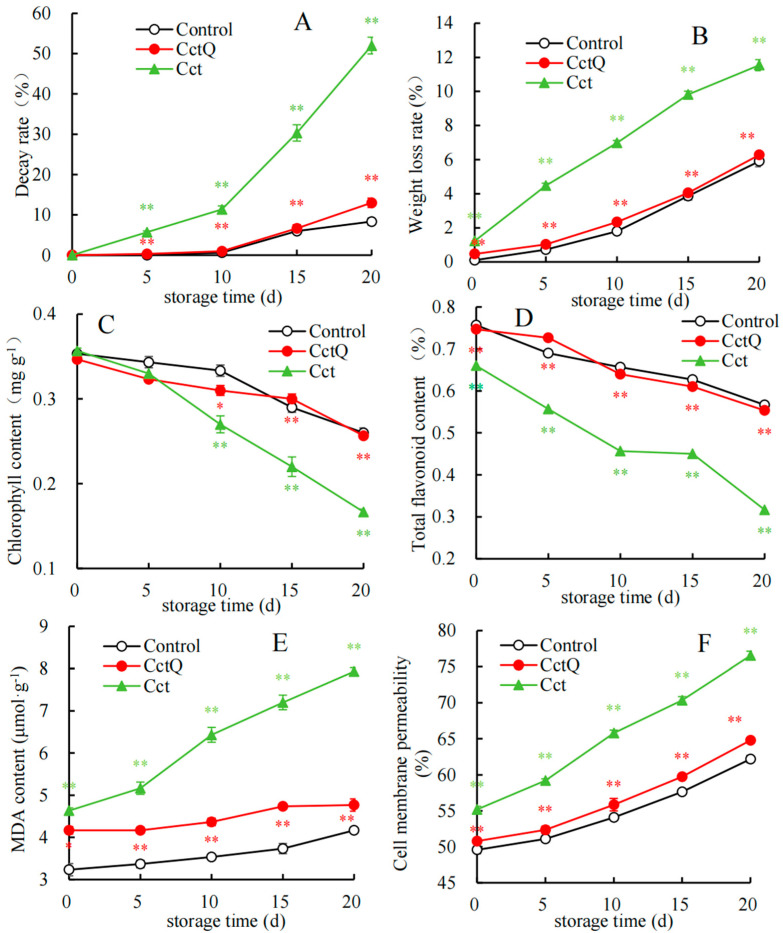
Effect of cushion packaging during cold chain transportation on (**A**) the delay rate, (**B**) weight loss rate, (**C**) chlorophyll content, (**D**) total flavonoid content, (**E**) MDA content, and (**F**) cell membrane permeability of Chinese olives during the shelf life. Asterisks indicate the significant differences between the Chinese olives invaded with unbuffered packing and the control fruit, with * and ** representing the significant levels at *p* < 0.01 or *p* < 0.05, respectively, on the same storage day. Symbols indicate the significant differences between the fruit treated with buffered packaging and the fruit subjected to unbuffered packaging, with * and ** denoting the significant levels at *p* < 0.01 or *p* < 0.05, respectively, based on the same shelf life.

During the shelf life, the decay rate and the weight loss rate exhibited a rising tendency in all three groups (Figure 1A,B). Compared with the control, a higher decay rate and weight loss rate was found in the unbuffering-packaged treated Chinese olives. There were remarkable differences in the decay rates within the shelf life of days 5–20 (*p* < 0.01), as well as in the weight loss rate between day 0 and 20. However, the decay rate and the weight loss rate in the buffer-packaged group were obviously lower than those in the unbuffering-packaged group within the shelf life (*p* < 0.01).

Figure 1C shows the changes in the content of chlorophyll, demonstrating an overall downward trend in all three groups. In comparison with the control group, the unbuffering-packaged treated group kept a lower chlorophyll content, with highly significant differences during the shelf life 10–20 (*p* < 0.01). However, the buffer-packaged group had a significantly higher chlorophyll content than the unbuffering-packaged-treated group within 10–20 d (*p* < 0.01). As exhibited in Figure 1D, the total flavonoid content in the three treatment groups gradually decreased with the extension of the shelf life. As storage continued, the unbuffering-packaged-treated fruits displayed a lower total flavonoid content than the control (*p* < 0.01), whereas total flavonoid amount in the buffer packing group was obviously higher compared to that of the unbuffering packaging group (*p* < 0.01).

During the entire shelf life, the MDA content (Figure 1E) and cell membrane permeability (Figure 1F) of Chinese olive fruits displayed a similar upward trend. Compared with control fruits, the unbuffering-packaged-treated group displayed dramatic increases, reaching a significant level (*p* < 0.01) throughout the shelf life. However, the MDA content and cell membrane permeability in the buffer-packaged group could keep lower values within the shelf life. Specifically, on day 20, the MDA content in the buffered-packaged group was almost half that of the unbuffered one. Moreover, on day 20, the cell membrane permeability was 1.2-fold lower than that of the unbuffered one.

### 3.2. Investigation of Antioxidative Enzyme Activities and Non-Enzymatic Antioxidant Contents in Chinese Olives

**Figure 2:**
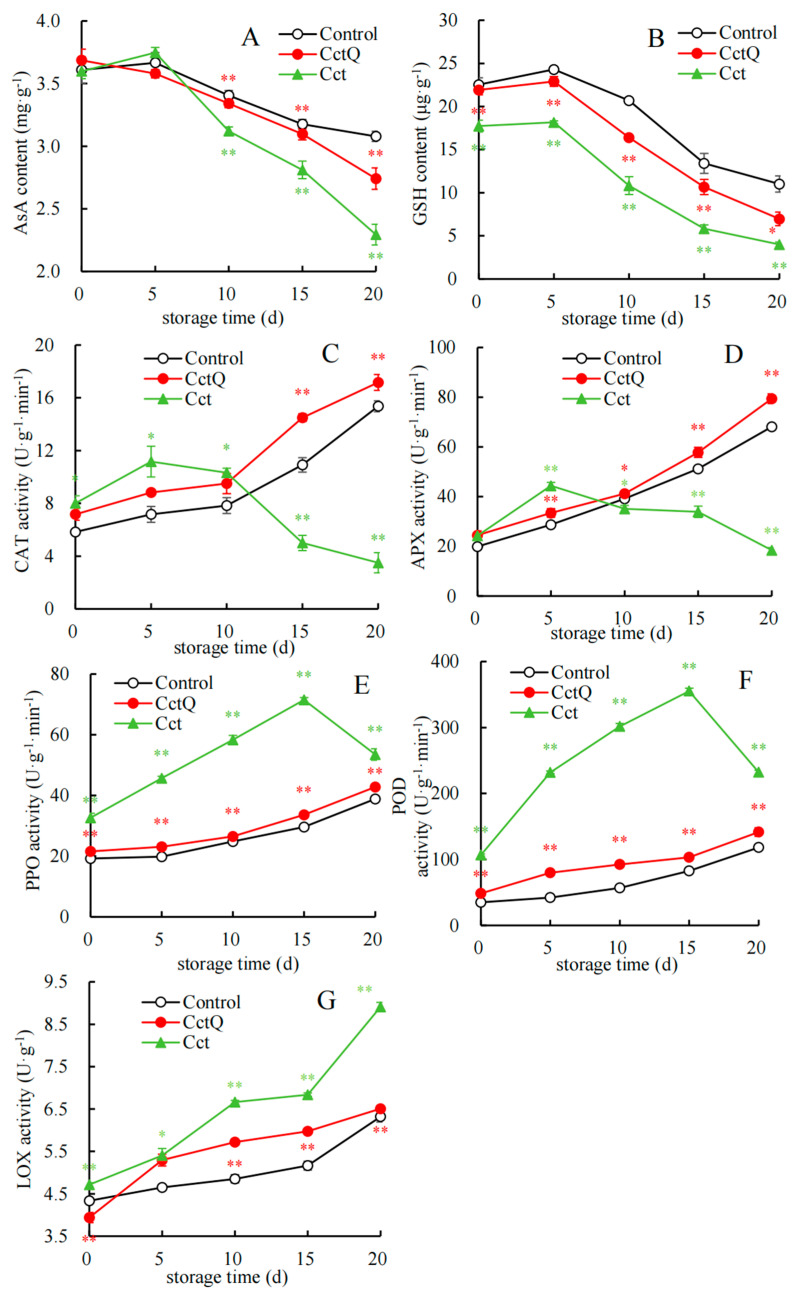
Effect of buffered packaging during cold chain transportation on AsA (**A**) and GSH(**B**) contents and activities of CAT (**C**), APX (**D**), PPO (**E**), POD (**F**), and LOX (**G**) in Chinese olives during the shelf life. The data presented in the figures are expressed as the mean ± SD of three biological replicates. Asterisks indicate the significant differences between the Chinese olives invaded with unbuffered packing and the control fruit, with * and ** representing the significant levels at *p* < 0.01 or *p* < 0.05, respectively, on the same storage day. Symbols indicate the significant differences between the fruit treated with buffered packaging and the fruit subjected to unbuffered packaging, with * and ** denoting the significant levels at *p* < 0.01 or *p* < 0.05, respectively, based on the same shelf life.

Overall, the non-enzymatic antioxidant content in all fruits showed a trend of first rising and then decreasing as shelf life progressed (Figure 2A,B). Among them, the unbuffering-packaged sample exhibited a significantly lower non-enzymatic antioxidant content than that of the control fruit, whereas the non-enzymatic antioxidant content of the buffering-packaged sample was noticeably higher than that in the unbuffering-packaged sample during the shelf life. A further study indicated that the clearly (*p* < 0.01) lower AsA content was detected in unbuffering-packaged Chinese olives than the control fruit on days 10–20, with these levels being 8.3%, 11.5%, and 25.5% lower than those in the control, respectively. In addition, the AsA content on days 10–20 in buffered packaging Chinese olives was 7.01%, 10.15%, and 19.49%, respectively, higher than the unbuffered packaging group. As for the GSH content, the unbuffering-packaged group showed a clearly (*p* < 0.01) lower level than the control group at the end of the shelf life (day 20), with these levels being 63.68% lower than those in the control. Thereafter, the GSH content on the last day of the shelf life in the buffering packaging Chinese olives was 74.23% higher than the unbuffered packaging group.

As depicted in Figure 2C,D, the CAT and APX activity in the unbuffered-packaged group increased slightly at the beginning of the shelf life (from day 0 to 5). Subsequently, it exhibited a descent from day 5 to 20, while the CAT and APX activity in both the buffered packaging treatment group and the control group showed a similar upward trend. During the initial shelf life (day 10), the CAT activity in the unbuffered packaging group was higher than that in the buffered packaging treatment group and the control group, with a notable disparity, but became lower than that of the other two groups on days 15–20. Furthermore, the APX activity in the unbuffered packaging group was higher than that in the buffered packaging treatment group and the control group in the early shelf life (days 0–5) but became lower than that of the other two groups on days 10–20.

Figure 2E,F show that the activity of PPO and POD in the three groups presented a rising trend on days 0–15; the unbuffered-packaged group reached peak activities of PPO and POD on day 15, with these levels being 2.41 and 4.3 times greater than those in the control, respectively, and then dropped sharply in the last period (15–20 d). Throughout the entire shelf life, the activities of PPO and POD enzymes in the buffer packing group were extremely lower than those in the unbuffered packing group (*p* < 0.01).

The LOX enzyme activity in all treatments presented an increasing tendency during the shelf life (Figure 2G). Additionally, the unbuffered packaging group had notably higher LOX enzyme activity than the control, while the buffer packing group distinctly retarded the increase in it. For instance, on the final day of the shelf life, the unbuffering-packaged group exhibited significantly higher level than the control group, with these levels being 41.00% higher than those in the control. Subsequently, the LOX enzyme activity of Chinese olives with buffering packaging on the last day of shelf life was 26.96% lower than that of the unbuffered packaging group.

### 3.3. Metabolic Profiles of Chinese Olive Fruits

**Figure 3:**
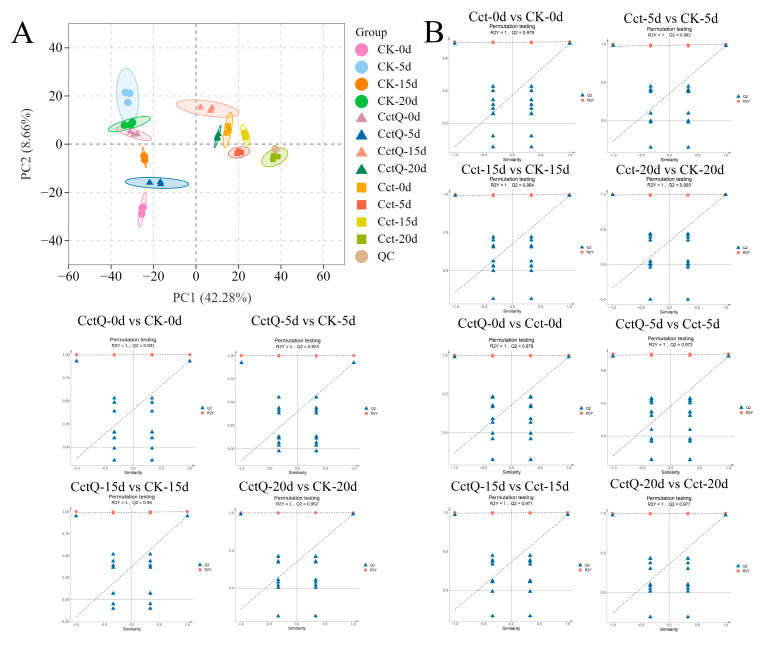
(**A**) The PCA shows the metabolic shift of Chinese olive fruits in different groups. Fruit samples of CK0d, CK5d, CK15d, CK20d, CctQ0d, CctQ5d, CctQ15d, CctQ20d, Cct0d, Cct5d, Cct15d, and Cct20d were mixed in equal volumes for use as the quality control (QC). (**B**) The OPLS-DA for different comparison groups.

To investigate the influence of cushioning packaging on the metabolome dynamic changes of Chinese olives during cold chain transportation, a PCA was conducted on all the detected metabolites. As shown in Figure 3A, the 36 samples from 12 groups clustered into 12 distinct areas on the plot, suggesting that the fruits at each stage of treatment and shelf life had relatively distinct metabolic profiles. As shown on the PC1, the control group (CK) and Cct treatment group were completely separated. Specifically, CK, CctQ 0d, and CctQ 5d were clustered in the left area, while the Cct treatment group, along with CctQ 15 d and CctQ 20 d, was clustered in the right area. Thus, these findings suggest that the metabolic profiling of the Cct treatment group differs significantly from that of the control. Moreover, the metabolic profiling of the CctQ treatment group is more similar to that of the control group in the early shelf life, while in the later shelf life, it bears much resemblance to that of the Cct treatment group. It appears that cushioning packaging may exert a significant impact on the non-volatile metabolites of Chinese olives. In addition, OPLS-DA models were conducted to screen DAMs among the three sample groups (Figure 3B). The Q^2^ value stands for the predictive capacity of the model, while R^2^X and R^2^Y denote the interpretation rate of the built model to the X-matrix and Y-matrix, respectively. As shown in Figure 3B, the three sample groups in the model were located within the confidence interval. At the same time, the parameters R^2^X and Q^2^ were both close to 1, indicating that the model was reliable. Thus, the OPLS-DA results indicate that statistically significant segregation occurred in the different comparison groups, used for further model testing and the selection of differential metabolites.

**Figure 4:**
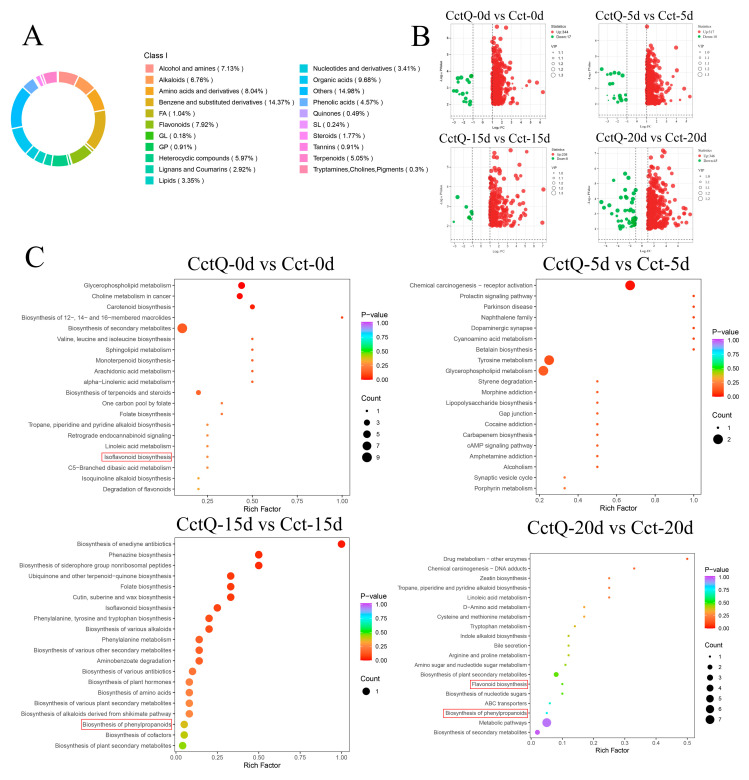
(**A**) Pie chart showing the different categories of metabolites identified in Chinese olives during the shelf life. (**B**) Volcano plots of the DAMs in CctQ-0d vs. Cct-0d, CctQ-5d vs. Cct-5d, CctQ-15d vs. Cct-15d, and CctQ-20d vs. Cct-20d. (**C**) KEGG enrichment analysis of differential metabolites in CctQ-0d vs. Cct-0d, CctQ-5d vs. Cct-5d, CctQ-15d vs. Cct-15d, and CctQ-20d vs. Cct-20d. (The red boxes indicated the pathways related to antioxidant capacity).

As shown in Figure 4A, the detected non-volatile metabolites were clustered into 21 categories during the shelf life. The largest number of metabolites belonged to other compounds (14.98%), followed by benzenes and their derivatives (14.37%), organic acids (9.68%), amino acids and their derivatives (8.04%), flavonoids (7.92%), alcohols and amines (7.13%), alkaloids (6.76%), heterocyclic compounds (5.97%), terpenoids (5.05%), phenolic acids (4.57%), nucleotides and their derivatives (3.41%), lipids (3.35%), lignans and coumarins (2.92%), steroids (1.77%), fatty acyls (1.04%), glycerophospholipids (0.91%), tannins (0.91%), quinones (0.49%), pigments (0.30%), sphingolipids (0.24%), and glycerolipids (0.18%). Then, the volcano graphs were generated to display the expression of all metabolites in fruits, in which various DAMs were identified during cold chain transportation and the shelf life (Figure 4B). To further confirm the metabolic profiles of cushioned packaging Chinese olives, attention was paid to the DAMs between the Cct-treated group and the CctQ-treated group.

The twenty most significantly enriched KEGG pathway entries in four groups (CctQ 0d vs. Cct 0d, CctQ 5d vs. Cct 5d, CctQ 15d vs. Cct 15d and CctQ 20d vs. Cct 20d) of fruits were selected, respectively. Figure 4C shows that DAMs were increased in the phenylpropanoid biosynthesis pathway and flavonoid biosynthesis pathway, suggesting that cushioning packaging is crucial for maintaining the antioxidant capacity of Chinese olive fruits during cold chain transportation.

### 3.4. Analysis of Key Metabolites of Pathways Related to the Antioxidant Capacity

**Figure 5:**
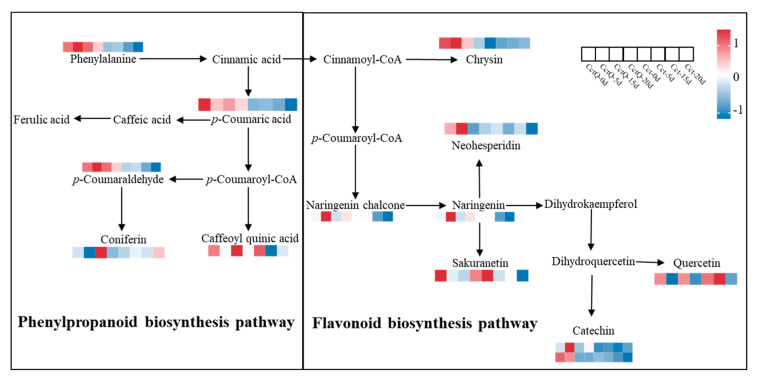
DAMs identified in the different comparison groups that are functionally associated with phenylpropanoid biosynthesis and flavonoid biosynthesis pathway.

Metabolic pathway analysis of flavonoid biosynthesis (ko00941) and phenylpropanoid biosynthesis (ko00940) suggested that the cushioning packaging treatment might affect the antioxidant capacity of Chinese olive fruits by affecting the accumulation of antioxidant metabolites (Figure 5). Thus, 12 metabolites, including 5 phenylpropanoid biosynthesis metabolites (phenylalanine, *p*-coumaric acid, *p*-coumaraldehyde, coniferin, and caffeoyl quinic acid) and 7 flavonoid biosynthesis metabolites (chrysin, sakuranetin, neohesperidin, naringenin chalcone, naringenin, catechin, and quercetin) were identified from all DAMs. In contrast to the unbuffered packaging group, the accumulation of all 12 antioxidant metabolites was significantly increased in cushioned packaging.

## 4. Discussion

Chinese olives are susceptible to mechanical damage during transportation, which can reduce their storability and lead to a decline in fruit quality. Currently, a research hotspot in the postharvest preservation technology of Chinese olives is focused on cold storage [23] and fresh-keeping agents [24], based on which it will suffer cold injury and potential compound residues, respectively. Consequently, this work investigates the cushioning packaging under cold chain transportation to prolong their shelf life.

### 4.1. Cushioning Packaging Influences the Post-Harvest Quality Parameters of Chinese Olives During the Shelf Life

The decay rate and weight loss rate serve as direct indicators of fruit quality during the shelf life, which are critical parameters for assessing the commodity value of the fruit [36,37]. In addition, changes in the peel color of fresh products were closely linked to commodity quality and were accompanied by changes in pigment content, including total flavonoid and chlorophyll [38]. Moreover, lipid peroxidation correlates with a compromised cell membrane integrity, while the accumulated malondialdehyde (MDA) content serves as an estimate of cellular membrane damage [39]. These parameters probably influence the postharvest fruit quality.

Our findings indicate that, in comparison to the control group, Chinese olives subjected to non-cushioning packaging exhibited a higher decay rate, weight loss rate, MDA content, and cell membrane permeability, alongside a lower total flavonoid content and chlorophyll content during the shelf life (Figure 1). In addition, cushioning packaging significantly reduced the decay rate and weight loss rate and effectively suppressed the reduction in total flavonoid and chlorophyll contents but maintained a lower MDA content and cell membrane permeability throughout the shelf life, compared with non-cushioning-packaging-treated fruits (Figure 1). Therefore, it could be inferred that cold chain transport may induce mechanical damage to fruits, resulting in the decreased shelf life quality and the cushioning packaging treatment could reduce the decay rate, weight loss rate, chlorophyll content, total flavonoid content, MDA content, and cell membrane permeability, thereby preserving the shelf life quality of Chinese olives.

### 4.2. The Impact of Cushioning Packaging on the Variations in Antioxidant Enzyme Activity and Antioxidant Substancs Contents in Chinese Olives Throughout the Shelf Life

ROS, as a crucial regulatory component in signal transduction, play a significant role in plant metabolism. However, the excessive generation of ROS can lead to cellular damage in plants. To prevent the accumulation of toxic byproducts associated with the high accumulation of ROS, each subcellular compartment within the organism is equipped with an antioxidant system that safeguards plant cell structures by scavenging ROS and maintaining redox homeostasis [23].

CAT and APX are protective enzymes in plants that rapidly mitigate the large bursts of ROS accumulation within cellular compartments. Specifically, CAT and APX collaborate to eliminate excess hydrogen peroxide (H_2_O_2_), converting it into H_2_O and O_2_, with APX functioning via the AsA–GSH cycle [40]. In this study, the unbuffered packaging treatment group exhibited an increase followed by a sharp decline in APX and CAT enzyme activity (Figure 2C,D). This trend may be attributed to changes in external environmental conditions prompting a stress response in fruits, which leads to elevated APX and CAT enzyme activities, thereby adapting to adverse conditions. Nevertheless, the large burst of ROS accumulation led to lipid peroxidation of cell membranes, disrupting the structural integrity and resulting in a significant decline in enzyme activity during the late stages of shelf life. Moreover, the trends and values observed in the buffer packaging treatment group were close to the control, suggesting that cushion packing can enhance CAT and APX activities during the shelf life, thereby effectively suppressing ROS accumulation. Similarly, H_2_O_2_-infected longan fruit exhibited elevated activities of SOD and CAT due to the oxidative burst triggered by the exogenous ROS. However, during the later storage stages, the activities of SOD and CAT in the H_2_O_2_-treated group decline, reducing their capacity to scavenge ROS [41]. The accumulation of ROS subsequently stimulates membrane lipid peroxidation, ultimately leading to a deterioration in fruit quality.

High levels of ROS can lead to lipid peroxidation within the cell membrane, consequently damaging the structural integrity of the membrane [42]. Lipoxygenase (LOX) is associated with membrane lipid peroxidation, which results in the degradation of plant cell membranes and potentially leads to a loss of cellular function [43]. The results of our study indicated that, in comparison to the control group, unbuffered packaging treatment significantly increased LOX enzymatic activity during the shelf life, whereas buffered packaging effectively delayed the rise in LOX activity (Figure 2G). Concurrently, lower levels of the MDA content (Figure 1E), cell membrane permeability (Figure 1F), and the decay rate (Figure 1A) were observed in buffered packing fruits. These findings indicate that buffered packaging treatment effectively mitigates the deterioration of fruit quality during cold chain transportation by alleviating the increase in LOX activity, inhibiting membrane lipid peroxidation, preserving the integrity of cell membrane structures, and consequently reducing the decay of Chinese olive fruits.

Phenolic compounds, PPO and POD, are localized in distinct cellular compartments of the plasma membrane. When the spatial barrier is disrupted, the phenolic compounds, PPO and POD interact rapidly, resulting in undesirable browning [44]. Our work demonstrates that throughout the entire shelf life, both buffered packaging and the control group effectively maintained lower levels of PPO and POD enzyme activity, whereas the unbuffered packaging group exhibited elevated levels (Figure 2E,F). Additionally, we observed that both the buffered packaging and the control group displayed a low level of cell membrane permeability, in contrast to the higher levels of cell membrane permeability in the unbuffered packaging group (Figure 1F). These findings suggest that damage to the cell membrane of unbuffered Chinese olive fruits resulted in the increased PPO and POD activity, thereby facilitating the oxidation of phenolic compounds. Conversely, the buffered packaging effectively preserved the integrity of cell membranes and inhibited oxidative processes, which is consistent with the lower levels of PPO and POD activity observed in this group. Likewise, Jiang et al. [45] demonstrated that the combination of 1-methylcyclopropene (1-MCP) and phytic acid effectively inhibited the accumulation of MDA, leading to a rapidly decreasing trend in the activities of PPO and POD and finally mitigating lipid peroxidation and delaying surface browning in fresh-cut peaches.

The AsA–GSH cycle, a crucial component of the non-enzymatic antioxidant system, plays a significant role in maintaining the redox state of plant cells. APX oxidizes AsA to dehydroascorbic acid while regulating reduced glutathione levels to scavenge superfluous H₂O₂ and protect cellular structures and function [46]. Research findings suggest that the AsA and GSH content in the unbuffered packaging treatment consistently exhibits a declining trend, significantly lower than that of the control group during the shelf life. However, buffered packaging treatment effectively delays the decline in AsA and GSH contents (Figure 2A,B). In addition, a reduced decay rate was observed in fruits with buffered packaging (Figure 1A). Consequently, it can be inferred that buffered packaging regulates both AsA and GSH contents, thereby reducing decay occurrence and preserving the shelf quality of Chinese olives.

### 4.3. Non-Targeted Metabolomic Analysis of Buffer Packaging Effects on the Changes in the Antioxidant Metabolite Contents of Chinese Olives During Shelf Life

The metabolome of fruits with unbuffered and buffered packaging was further analyzed by using LC-MS. A large number of DAMs were detected, demonstrating that cushioning packaging could induce substantial changes in metabolite levels. KEGG pathways, which categorized DAMs into different pathways, provided valuable taxonomy for studying complex biological functions (Figure 4B). Here, KEGG enrichment analysis (Figure 4C) revealed that DAMs were significantly enriched in the phenylpropanoid and flavonoid biosynthesis pathways (*p* < 0.05), demonstrating that cushioning packaging treatment modulated these pathways. We performed a comprehensive analysis of the two differential metabolic pathways and found that the phenylpropanoid and flavonoid biosynthesis pathways may be primary contributors to the observed differences in antioxidant activity among Chinese olives.

In response to external stress, plants exhibit the significant accumulation of phenylpropanoid metabolites, which are essential secondary metabolites that enhance resistance by rendering the cellulose in the cell wall against degradation during various biotic or abiotic challenges [47]. Choo et al. [48] reported that the contents of flavonoids, phenylpropanoids, fatty acids, and terpenoids were significantly increased in barley with resistance during the infection of *Fusarium* head blight and mycotoxin. Liu et al. [49] demonstrated that mulberry trees are abundant in phenylpropanoids, alkaloids, and other metabolites that play a critical role in plant responses to various external stress. Furthermore, phenylalanine functions as a substrate in primary and secondary metabolic pathways and serves as a precursor to various phenolic compounds closely associated with plant resistance. Exogenous phenylalanine can regulate plant growth, photosynthesis, and antioxidant defense mechanisms, thereby mitigating drought stress in mustard [50]. Our study found that a significant upregulation of phenylalanine, *p*-coumaraldehyde, *p*-coumaric acid, coniferin and caffeoyl quinic acid metabolites within the phenylalanine metabolic pathway was observed in Chinese olive fruits subjected to buffer packaging treatment, thereby indicating that buffer packaging may enhance the antioxidant processes in fruits by promoting the accumulation of these five DAMs (Figure 5). Flavonoids, a class of natural products widely distributed in plants, exhibit remarkably significant antioxidant activity. The relationship between flavonoids in various sugarcane varieties and antioxidant activity has been elucidated using a widely targeted LC-MS/MS approach, indicating that higher levels of flavonoids are closely linked to greater antioxidant capacity, which can resist the oxidative stress induced by diverse abiotic factors [51]. In our study, chrysin, neohesperidin, naringenin chalcone, sakuranetin, quercetin, catechin, and naringenin metabolites within the flavonoid biosynthesis pathway were notably upregulated. Consequently, we propose that the enhancement of antioxidant activity in Chinese olive fruits following buffer packaging treatment is closely associated with the accumulation of flavonoid metabolites.

## 5. Conclusions

In conclusion, our current research elucidates the mechanisms by which buffer packaging treatment alleviates the deterioration of Chinese olive fruit quality during cold chain transportation, primarily encompassing the following aspects: (1) buffer packaging treatment effectively retards increases in the decay rate, weight loss rate, MDA content, and cell membrane permeability. Simultaneously, it delays the decline in chlorophyll and total flavonoid contents, thereby maintaining the overall quality of the fruits during the shelf life after cold chain transportation. (2) Buffered packaging treatment exhibits elevated activities of antioxidant enzymes (APX and CAT) alongside the retardation of the reduction of ASA and GSH contents, which enhanced the antioxidant capacity of the fruits. (3) The buffered packaging group exhibits lower levels of POD, PPO, and LOX activity, thereby reducing membrane lipid peroxidation and stabilizing the integrity of cell membranes. (4) Buffered packaging treatment leads to the accumulation of the phenylpropanoid pathway metabolites (phenylalanine, *p*-coumaraldehyde, *p*-coumaric acid, coniferin, and caffeoyl quinic acid) and flavonoid biosynthesis pathways metabolites (chrysin, neohesperidin, naringenin chalcone, sakuranetin, quercetin, catechin, and naringenin) during the shelf life, which are correlated with antioxidant activity. These findings provide a solid theoretical framework for further exploring how buffer packaging treatment enhances the antioxidant capacity of Chinese olives and alleviates postharvest quality declines and offer insights into strategies for extending their shelf life.

## Data Availability

The original contributions presented in this study are included in the article/Supplementary Materials. Further inquiries can be directed to the corresponding author.

## Supplementary Materials

The following supporting information can be downloaded at: https://www.mdpi.com/article/10.3390/foods13244133/s1, Figure S1. Packaging materials employed during cold chain transportation (A) aluminum film cold chain carton, (B) air column bag cushioning packaging material (C) polythene film bag.
