# Supplementary material for: Investigation of Effects of Cushioning Packaging on the Physiological and Quality Changes in Chinese Olive Fruits During Cold Chain Transportation
Source: Foods. 2024 Dec 20;13(24):4133. doi: 10.3390/foods13244133 (PMC11675954; doi:10.3390/foods13244133)
Supplement: Supplementary file 1 [file foods-13-04133-s001.zip › foods-3339217-supplementary.pdf]

Supplementary Materials

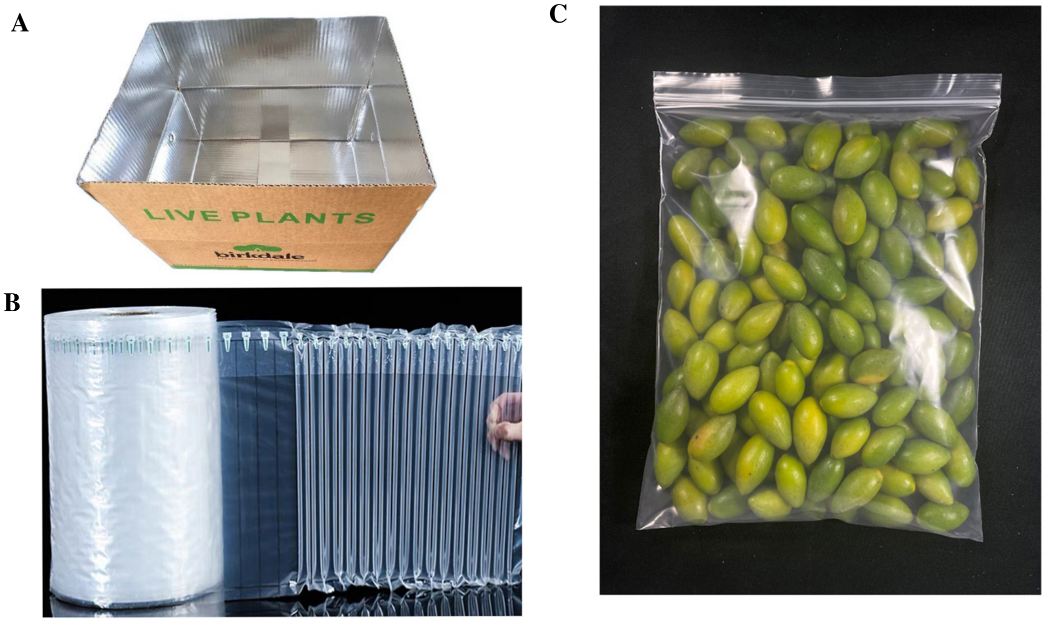

Figure S1. Packaging materials employed during cold chain transportation (**A**) aluminum film cold chain carton, (**B**) air column bag cushioning packaging material (**C**) polythene film bag.
